# When I say … absenteeism

**DOI:** 10.1111/medu.70212

**Published:** 2026-03-24

**Authors:** Lorenzo Madrazo, Samantha Halman, Susan Humphrey‐Murto, Kori A. LaDonna

**Affiliations:** ^1^ Department of Medicine University of Ottawa Ottawa Ontario Canada; ^2^ Department of Innovation in Medical Education University of Ottawa Ottawa Ontario Canada

## Abstract

In this When I Say… article, the authors invite us to reconsider how we interpret absenteeism, reframing it as a reflection of our shared culture rather than not individual failure. *#MedEd*

## 1 INTRODUCTION

Physician and resident absences from work have become a growing concern in recent years, particularly as the profession has become more attuned to issues of physician well‐being and faces lingering effects from the COVID‐19 pandemic.^1^, ^2^ ‘Absenteeism’ has been used in the literature to encompass all forms of absences.^3^, ^4^ Yet in a culture that values presenteeism, or coming to work even when sick,^5^ the word absenteeism does more than describe—it passes judgement, carrying implicit assumptions about character, commitment and professionalism. As we unpack throughout this article, we suggest that not all forms of absence are equal and argue that not all absences connote absenteeism. A more critical examination of how absenteeism is defined—and of what is implied when the term is used—may help advance more nuanced and productive conversations about physicians' work and well‐being.

## 2 CONCEPTUALISING ABSENCE AND ABSENTEEISM

We define *absence* as a discrete event—someone is away from rounds, a call shift, clinic or a mandatory teaching session. Absences can either be *planned* (e.g., vacations, parental leave or attendance at conferences) or *unplanned* (e.g., sudden illness, personal emergencies or urgent family responsibilities). Planned absences are usually less controversial because they are expected. In contrast, unplanned absences disrupt work, inconvenience patients, and thus draw more scrutiny, often prompting questions of legitimacy even when fully justified.^1^, ^2^ Among unplanned absences, none invite more suspicion than those deemed avoidable or unsanctioned^1^, ^2^—what the education literature calls *truancy*.^3^ In practice, an example of truancy would be the physician or trainee who ‘calls in sick’ but later posts about their presence at a personal event on social media. Although truancy entails deliberate deception, not all unplanned absences are dishonest.

In contrast to its colloquial use, we conceptualise *absenteeism* not as a single absence—or even a tally of absences—but as absences interpreted as a pattern of behaviour, often experienced as the internal voice that asks, ‘Dr. X is away *again?*’ While patterns of absenteeism have been linked with problems such as burnout, career dissatisfaction and substance use, not every unplanned absence signals such problems.^6^ We therefore emphasise absenteeism as an *interpretation* rather than direct observation: We imply a pattern from what we see, recognising that we do not see or know everything. Yet despite the many possible explanations for absence, our interpretations are rarely generous: Interpretation quickly becomes judgement and judgement a proxy for character.

## 3 THE DEMERITS OF ABSENTEEISM

Why does this matter? Because labels stick. In medicine, presence is currency. Those engaged in presenteeism are seen as ‘dedicated’ and accrue social capital despite other personal costs. In contrast, even a legitimate but poorly timed absence risks reputational demerit, marking a resident or faculty member as ‘unreliable’. Consider this: When a colleague calls in sick the day after vacation, do we extend grace—or are we more prone to suspect impropriety? Such moments expose a culture where trust is provisional and must be continually earned through presence. Thus, each unplanned absence risks compromising one's ability to maintain a socially desirable image—particularly when legitimate absences are reflexively misread as a pattern of truancy or laziness. Residents are acutely aware of these dynamics, often hesitating to call in sick to either avoid burdening colleagues or to protect their reputations.^1^

The point is not to ignore patterns of behaviour that could signal wellness or professionalism issues. However, we should resist making truancy the ‘knee‐jerk’ assumption, as not every absence is evidence of deeper dysfunction or nefarious intent. Thoughtful and compassionate discernment is required to distinguish between legitimate needs for time away and indications that a colleague may be struggling or acting unprofessionally. Making such distinctions with precision is often difficult, clouded by limited information and the social discomfort of confronting peers. Further complicating matters, perceptions of what makes an absence ‘legitimate’ are anything but universal. Even within our author team, different positionalities shaped how we interpreted the legitimacy of faculty and trainee absences, underscoring that views of professionalism are fluid and contextual.

Incorrect assumptions have consequences. A recent study found that residents with *any* unplanned on‐call absences were less likely to receive academic recognition,^6^ suggesting that absence might influence judgements on professionalism and merit. Thus, absenteeism does not just describe a perceived pattern of absence; it can actively shape how residents are recognised and rewarded within the culture of medicine.

Physicians and trainees who parent or who live with chronic illness or disability are particularly vulnerable to these demerits, as they may legitimately require more time away from work.^7^, ^8^ Although sharing why you were absent may invite empathy that legitimises the absence, stigma can make such disclosures difficult. Consequently, those left to absorb the impact of an absence seldom know its full context, inviting misinterpretations shaped by individual biases and ableist structures that continue to disadvantage those with chronic illness or disability.^8^

## 4 IMPLICATIONS FOR EDUCATORS

Taken together, these dynamics reveal how presenteeism and absenteeism are often cast as individual virtues or failings. But this moral framing draws attention away from structural and cultural influences that shape them—heavy workloads, lack of sick leave, inflexible staffing systems, inadequate remuneration models and the unspoken expectation that doctors should be ‘selfless superhumans’.^5^, ^8^

Educators and healthcare leaders must therefore not only challenge their assumptions but also take practical steps to reshape the conditions that make absence feel morally charged—reducing the perceived tension between caring for patients, protecting colleagues from additional burden, and caring for oneself. This includes reviewing and clarifying absence and back‐up policies so expectations are explicit, transparent and do not hinge on informal norms around ‘toughness’. We must also monitor workload patterns and coverage systems not only to distribute strain fairly but to identify and reform structural features that produce repeated activation and uneven burden—conditions that both reflect and exacerbate burnout.^1^ Finally, we should examine how remuneration models and performance expectations may unintentionally reward overwork and endurance over sustainability. These efforts must extend across the continuum of medical education—from medical students to residents to attending physicians—so that consistent norms are reinforced and meaningful culture change becomes possible.

As we carry this conversation forward, we look toward a time when saying ‘absenteeism’ stops being about *them*—a reflection of individual reliability—and becomes about *us*—a reflection of how we care for ourselves collectively.

## AUTHOR CONTRIBUTIONS

**Lorenzo Madrazo:** Conceptualization; writing – original draft; writing – review and editing. **Samantha Halman:** Writing – review and editing; conceptualization. **Susan Humphrey‐Murto:** Writing – review and editing; conceptualization. **Kori A. LaDonna:** Conceptualization; writing – review and editing.

## Data Availability

Data sharing is not applicable to this article as no datasets were generated or analysed during the current study.
